# Outcomes of a Clinical Pathway for Pleural Disease Management: “Pleural Pathway”

**DOI:** 10.1155/2018/2035248

**Published:** 2018-04-01

**Authors:** Srinivas R. Mummadi, Peter Y. Hahn

**Affiliations:** Department of Pulmonary and Critical Care Medicine, Metro Health-University of Michigan Health, Suite 220, 2122 Health Dr. SW, Wyoming, MI 49519, USA

## Abstract

**Background and Objectives:**

Clinical pathways are evidence based multidisciplinary team approaches to optimize patient care. Pleural diseases are common and accounted for 3.4 billion US $ in 2014 US inpatient aggregate charges (HCUPnet data). An institutional clinical pathway (“pleural pathway”) was implemented in conjunction with a dedicated pleural service. Design, implementation, and outcomes of the pleural pathway (from August 1, 2014, to July 31, 2015) in comparison to a previous era (from August 1, 2013, to July 31, 2014) are described.

**Methods:**

Tuality Healthcare is a 215-bed community healthcare system in Hillsboro, OR, USA. With the objective of standardizing pleural disease care, locally adapted British Thoracic Society guidelines and a centralized pleural service were implemented in the* “pathway”* era. System-wide consensus regarding institutional guidelines for care of pleural disease was achieved. Preimplementation activities included training, acquisition of ultrasound equipment, and system-wide education. An audit database was set up with the intent of prospective audits. An administrative database was used for harvesting outcomes data and comparing them with the* “prior to pathway”* era.

**Results:**

54 unique consults were performed. A total of 55 ultrasound examinations and 60 pleural procedures were performed. All-cause inpatient pleural admissions were lower in the “pathway” era (*n* = 9) compared to the “prior to pathway” era (*n* = 17). Gains in average case charges (21,737$ versus 18,818.2$/case) and average length of stay (3.65 versus 2.78 days/case) were seen in the “pathway” era.

**Conclusion:**

A “pleural pathway” and a centralized pleural service are associated with reduction in case charges, inpatient admissions, and length of stay for pleural conditions.

## 1. Introduction

Clinical pathways have been described as evidence based multidisciplinary team approaches to optimize patient care for a presenting clinical diagnosis [1, 2]. Salutary effects on patient outcomes, length of stay, and hospital costs have been observed with implementation of clinical pathways [3]. Pleural diseases are common and accounted for 3.4 billion US $ in 2014 US inpatient aggregate charges (“National Bill” as estimated by HCUPnet, a query system based on Healthcare Cost and Utilization Project [HCUP] data) [4]. Due to wide variation in practice styles for managing initial manifestations of pleural disease [5, 6], an institutional clinical pathway (“pleural pathway”) adapted from the British Thoracic Society (BTS) pleural disease guidelines [7–11] was implemented in conjunction with a dedicated pleural service [12, 13]. We hypothesized that improved inpatient outcomes (lower average length of stay, charges, and admissions) would have resulted due to the implementation of a pleural pathway. A narrative on the design, implementation, and outcomes of the pleural pathway in comparison to the prior era is provided in this manuscript. Some of the results of this study were previously reported in the form of an abstract [14].

## 2. Methods

### 2.1. Design

A descriptive observational study design was used for studying outcomes of a clinical pathway and a centralized pleural disease service in a community healthcare system.

### 2.2. Setting

Tuality Healthcare (An Oregon Health & Science University [Portland, OR] partner) is a 215-bed healthcare system in Hillsboro, OR, USA. It is comprised of two hospitals and additional outpatient treatment facilities. Outpatient and inpatient consultative services for pulmonary disease are provided by pulmonary consultants and thoracic surgeons with procedural support from radiology. Pleural disease management prior to the introduction of the pleural pathway was provided using the following modalities:

1. Large spontaneous pneumothoraces: emergency room (ER) insertion of large bore chest tubes (>14 F) was followed by inpatient admission and general/thoracic surgery consultation.
2. Unilateral pleural effusions presenting with mediastinal shift were admitted to the hospital. Stable unilateral pleural effusions were aspirated in the scheduled outpatient radiology setting and followed by the referring physician.
3. Non-ultrasound-guided management of pleural diseases was carried out in the pulmonary clinic.
4. Prolonged air leaks (secondary spontaneous pneumothorax, postoperative) were managed with watchful waiting and if needed via surgical intervention by thoracic surgery.
5. Fibrinolytics (tPA + DNase) were not used in management of empyema.

In May 2014, an institutional need was felt for standardizing pleural disease care in response to observed institutional variation in pleural care and low performance on a publicly reportable healthcare quality measure (Patient Safety Indicator # 6 measuring rates of iatrogenic pneumothorax). In response to the need, locally adapted BTS 2010 pleural guidelines [7–11] were devised by the author (SM). Two thoracic ultrasound and pleural procedures, trained pulmonary physicians, and a nurse practitioner constituted the pleural service. Key deviations from the BTS guidelines included using noncontrast CT scan for further diagnostic imaging of an aspirated unilateral pleural effusion, narrow bore (8 F) chest tube and Heimlich valve insertion for outpatient management of primary spontaneous pneumothorax, and use of intrabronchial valves for prolonged air leaks (see Supplementary Materials (available here)). When indicated, pleuroscopic examination and parietal pleural biopsies were performed by a pulmonary physician aided by an anesthesiologist administered moderate sedation. Overnight hospital observation was performed after pleuroscopy procedure. Gravity assisted thoracentesis drainage was adopted institutionally.

A system-wide consensus was generated via multidisciplinary efforts across diverse division meetings (Emergency Department, Pulmonary & Hospital Medicine, General & Thoracic Surgery, Interventional Radiology, Primary Care) over a span of 3 months. Approvals for institutional guidelines emphasizing patient safety (ultrasound assistance for pleural procedures in the nonradiology setting [15, 16], nurse driven “pleural checklists” [17]) and turnaround time (inpatient pleural consults answered within 24 hours; 72 hours for outpatients palliated in the ER and assured follow-up in the pulmonary clinic for discharged patients) were sought and buy-in was secured from all the stakeholders. Algorithms for management of unilateral pleural effusions (exudative, transudative, malignant, hemothorax, empyema) and spontaneous pneumothoraces (8 F chest tube coupled with a self-contained Heimlich valve apparatus as the primary modality) were approved and disseminated throughout the institution. Shift towards ambulatory care of unilateral pleural effusions was promoted to primary care teams in monthly primary care administrative meetings. Admission avoidance was stated as an intended benefit. In preparation, pleural supply carts (narrow bore chest tubes [8 F], medium bore chest tubes [14 F], Heimlich valves, thoracentesis kits) were designed and standardized across multiple locations (pulmonary clinic, ER, and the intensive care unit). Ultrasound machines with high and low frequency probes were already available in the ER and intensive care unit. Pulmonary clinic acquired a new low frequency probe and shared the existing ultrasound machine/high frequency probe with endocrinology clinic. In addition, minimally invasive interventions for prolonged air leaks (bronchoscopic insertion of IBV® valves) were promoted institutionally. Funding support was obtained from the hospital and aided by building a business case centered on cost effectiveness of coordinated pleural care, usage of ultrasound, and potential to improve upon the publicly reported quality measures. Preimplementation system-wide education [7] (didactic lectures, simulated and supervised practice of chest tube care, fibrinolytic installation) was completed for all the nursing units involved in the care of pleural disease patients before the “go-live” date.

Outpatient and inpatient pleural consults were channelled via a “pleural pathway” with the pleural consultative service as the cornerstone starting on August 1, 2014.

An audit database (Microsoft Excel, Redmond, Washington, USA) was set up with the intent of prospective audits and capturing data about patients seen through this pathway. Database management support was provided by a trained medical assistant. A one-year audit was performed by comparing outcomes data from the “pathway” era (from August 1, 2014, to July 31, 2015) with a “prior to pathway” era (from August 1, 2013, to July 31, 2014). Outcomes data such as number, type of diagnostic and therapeutic procedures, ultrasound examinations performed, and adverse events were restricted to pleural pathway patients and housed in the audit database.

Data for average case charge (US $), average length of stay (LOS) in days, and number of inpatient admissions in both eras were extracted utilizing the visual analytic tools of a subscription service focusing on care variation (Crimson Clinical Advantage Continuum of Care, Release CCC.2016.09. Isaac Newton, Washington, DC, USA). These data are derived from coding and billing records of the institution and are restricted to inpatients. The following diagnosis-related group (DRG) codes [18] were used to define pleural diseases during the analysis: 187 (pleural effusion* with* complication or comorbidity [CC]), 188 (pleural effusion* without* complication or comorbidity [CC] and major complication or comorbidity [MCC]), 199 (pneumothorax* with* MCC), 200 (pneumothorax* with* CC), and 201 (pneumothorax* without* CC and MCC). Examples of conditions that define major comorbidity (MCC)/comorbidity (CC) are provided in footnotes of Table 2.

Information regarding annual number of inpatient admissions and ER visits was obtained from the Department of Finance's comparative statistics detail report. An exemption for institutional review board's review and need for individual consent was granted by Western IRB (submission tracking number 1-900642-1).

## 3. Results

Hospital recorded 3,549 inpatient admissions in the “prior to pathway” era and 3,154 inpatient admissions in the “pathway” era. Emergency room (ER) recorded 31,232 visits in the “prior to pathway” era and 32,688 visits in the “pathway” era.

Audit findings during the pathway era are described in Table 1. 54 unique pleural consults spanning over ER, inpatient and outpatient domains, were provided during the pathway era. All the consults were performed as stated in the institutional guidelines (i.e., inpatient pleural consults answered within 24 hours; 72 hours for outpatients palliated in the ER and assured follow-up in the pulmonary clinic for discharged patients). There was no documented follow-up for 7 consultations due to patients' preferences. Complications recorded in the audit are as follows: pain during fluid drainage phase of thoracentesis (*n* = 5), iatrogenic pneumothoraces (*n* = 2), chest drain dislodgement (*n* = 3). One case of subcutaneous emphysema was recorded in the audit. Additional information regarding complications is provided in Table 1.

Outcomes data for pleural conditions are presented in Table 2. Overall, all-cause pleural inpatient admissions were lower in the pathway era (17 in the “prior to pathway” era versus 9 in the “pathway” era). This was accompanied by lower average case charges (21,737 versus 18,818.2$) and lower average length of stay (3.65 versus 2.78 days).

While pleural effusion with significant comorbid conditions saw a decrease in charges, there was no observed decrease in LOS and inpatient admissions. Pleural effusion with no associated comorbidities saw an increase in case charges with no change in LOS.

Pneumothorax irrespective of associated comorbidity saw a decrease in the number of inpatient admissions, length of stay, and average case charges.

## 4. Discussion

This study is the first in North America describing a dedicated ambulatory/inpatient pleural clinical pathway and associated gains in the efficiency domain of healthcare quality [19]. Recent developments in advancing the diagnosis and management of pleural disease call for a redesigned care model in tune with the modern definition of healthcare quality [19]. Emergency admissions for pleural effusions are recognized as a contributor to the inefficiency of healthcare [20] and are a target for admission avoidance in the UK [13]. Outpatient treatment of primary spontaneous pneumothoraces using an 8 F narrow bore chest tube and Heimlich valve has been shown to be safe and cost effective [21, 22]. Intrapleural t-PA and DNase have been shown to improve the drainage, length of stay, and the frequency of surgical intervention in empyema. Indwelling pleural catheters have replaced repeated thoracentesis as the primary modality for management of malignant pleural effusions [23, 24]. Despite the recognition of pleural medicine as a distinct subspecialty, a recent audit in the UK found that only 21% of facilities (serving 33 million patients) incorporated a specialist pleural disease clinic [25]. Changes in institutional practice guidelines augmented by education [26] and audit have been shown to positively impact the outcomes in pleural disease [27].

In our analysis, charges for pleural effusion care increased and were offset by a reduction in pneumothorax charges. In 1996, a small randomized trial found similar savings with a narrow bore chest tube insertion and ambulatory management of spontaneous pneumothorax patients (cost savings of 5660 US$/patient and savings of 5 bed days per case) [28]. While definite attribution is difficult, it is likely that the usage of moderate sedation and overnight in-hospital observation for pleuroscopy cases (performed for undiagnosed exudative pleural effusions) have played a role in the increase of pleural effusion costs. Recently a multicenter experience reported local anesthetic pleuroscopy with the same day discharge as safe, effective, and efficient suggesting further room for cost savings [29].

Resources are needed for a functional pleural pathway including training, acquisition of ultrasound machines, and associated probes housed in nonradiology settings. However, it is likely that such investment yields returns by enhancing timely and safe care [30]. Delay in acquiring outpatient ultrasound scans due to radiology scheduling load contributes to costs of 24,890 US $ per annum [30]. A non-ultrasound-guided thoracentesis complicated by a pneumothorax increases the cost of hospitalization by $ 2,801 and increases the length of stay by 1.5 days [31]. Costs for iatrogenic pneumothorax escalate once the patient is hospitalized [32]. It is essential that physician champions utilize such data in their funding requests to the senior leadership. An important component of this pathway was the role of ER physicians adopting the institutional guidelines for the management of unilateral pleural effusions and spontaneous pneumothoraces in achieving the goals of this pathway [33]. This is a crucial factor in determining the success of a pleural pathway.

Complications observed in our series deserve further attention. 7.3% of inserted drains fell out in a recent British audit highlighting the need for improvement in securement strategies [25]. 14% (3/21 insertions) of chest drains dislodged in our audit suggesting that it is an important area for further quality improvement.

There are several methodological limitations to our study. Administrative databases were used for cost effectiveness metrics and were restricted to inpatients. Inpatient charges were utilized as surrogate for costs. Information was lacking about outpatient charges including radiology procedures. It is also possible that not all patients presenting with pleural conditions were channelled via the pleural pathway. Audit data were not available on patients outside the pathway during the study period. A cost benefit analysis incorporating input costs was not performed. Lack of propensity matching and an interrupted-time series design impacts the validity of these findings. We acknowledge that our series has a relatively small sample size and is based on a retrospective and limited time frame of observation. Lack of control cohorts in the baseline and the intervention period is a significant limitation. However, no prior attempts exist in reporting improvements in processes/outcomes after implementation of coordinated pleural care. We believe our study should be viewed as an attempt in stimulating further research in this field.

In conclusion, we report the evolution of a clinical pathway and a centralized pleural service for the care of pleural disease in a community healthcare system. Lower case charges and length of stay were observed for inpatient pleural conditions, largely mediated by strides in pneumothorax management. However, these findings need further study.

## Figures and Tables

**Table 1:** Key findings from the audit during the pathway era (from August 1, 2014, to July 31, 2015).

Characteristics	Results
Age at evaluation	63 (56–75)
Median (IQR)—yr.	
Female sex—no. (%)	15 (40.5)
Unique consults^*∗*^—no.	54
Initial consult location—no. (%)	
Emergency department	12 (22.2)
Outpatient	20 (37)
Inpatient	22 (40.7)
Consults with documented follow up—no. (%)	47 (87)
Ultrasound examinations—no.	55
Primary pleural diagnosis—no.	40
Primary spontaneous pneumothorax	3
Secondary spontaneous pneumothorax	5
Traumatic pneumothorax	4
Noninfectious and nonmalignant exudative effusions	7
Empyema^∂^	3
Paramalignant effusion	4
Malignant pleural effusion	5
Transudate effusions	5
Others (giant bullae, indwelling IPC s/p pleurodesis, normal pleural ultrasound exam)	4
Pleural procedures—no.	60
8 F chest tube insertion^*π*^	6
14 F chest tube insertion	4
>14 F chest tube insertion^†^	8
Thoracentesis	26
Indwelling pleural catheter (IPC) insertion	3
IPC removal^*Ω*^	1
Pleuroscopy (with and without pleural biopsy)	3
IBV® valve insertion^ß^	3
VATS assisted bleb resection^Δ^	3
Surgical decortication	3
Complications—no.	14
Pain during fluid drainage	5
Iatrogenic pneumothorax^¶^	2
ER evaluation due to patient concerns^§^	2
Chest drain dislodgement^‡^	3
IPC track metastasis	1
Subcutaneous emphysema	1

^*∗*^8 patients had more than one unique consult due to recurrent disease on the same or contralateral side. There were 37 unique patients in the audit. ^∂^These patients received initial fibrinolytic treatment (tPA + DNase). ^*π*^8 F tube with an inbuilt Heimlich valve apparatus. ^†^Two insertions were in the setting of secondary spontaneous pneumothoraces with acute respiratory failure and one insertion to palliate a concurrent large pleural effusion and an iatrogenic pneumothorax. Five insertions were in the postoperative setting; ^Ω^Removed in the ER (after pleurodesis confirmation) in the context of a Health Information Exchange alert about multiple area ER visits for a “nonfunctional catheter.” ^ß^IBV Valve (Spiration, Redmond, WA, USA) is a unidirectional valve that blocks air entry distally and is inserted via a bronchoscopic procedure. In our series, all of them were inserted in prolonged air leaks due to secondary spontaneous pneumothoraces. ^Δ^Video assisted thoracoscopy. ^¶^One of the episodes required an ambulatory 8 F chest tube. 2nd episode required hospital admission due to lack of credible follow-up. ^§^Emergency room evaluation related to patient concerned about serosanguinous discharge into the ambulatory 8 F chest tube. ^‡^None of the dislodged chest tubes required reinsertion.

**Table 2:** Outcomes data for inpatient pleural care (DRG codes) during the two eras: “prior to pathway” versus “pathway.”

Pleural condition	Inpatient admissions in prior to pathway era	Inpatient admissions during pathway era	Average LOS in prior to pathway era (days)	Average LOS during pathway era (days)	Average case charges in prior to pathway era ($)	Average case charges during pathway era ($)
Pleural effusion with MCC^¥^ & CC^∞^	4	3	3.00	3.00	22,884.7	17,092
Pleural effusion without MCC^¥^ & CC^∞^	3	3	2.67	2.67	12,874.3	24,081.6
Pneumothorax with MCC^¥^	3	0	5.67	0	36,637.3	0
Pneumothorax with CC^∞^	5	2	2.80	3.00	18,706.6	14,277.5
Pneumothorax without CC^∞^ & MCC^¥^	2	1	5.50	2.00	17,967	11,563
Overall pleural conditions	17	9	3.65	2.78	21,737	18,818.2

^*¥*^MCC: major complications/comorbid conditions such as congestive heart failure, stroke, coma, acute MI, HIV, acute respiratory failure, and cardiac arrest. ^*∞*^CC: complications/comorbid conditions such as angina, delirium, dementia, anemia, cachexia, and COPD with exacerbation.

## Additional Points

*Summary at a Glance. *A descriptive narrative of a clinical pathway for pleural disease is the focus of this manuscript. Administrative data (charges, length of stay) were examined in* “before”* and* “during”* clinical pathway eras. Our study offers a real-life model of efficient pleural disease care.

## Supplementary Materials

Supplementary Materials File 1: algorithm for institutional management for symptomatic pneumothorax in adult patients (≥18 yrs. of age).
