# Supplementary material for: Outcomes of a Clinical Pathway for Pleural Disease Management: “Pleural Pathway”
Source: Pulm Med. 2018 Apr 1;2018:2035248. doi: 10.1155/2018/2035248 (PMC5899858; doi:10.1155/2018/2035248)
Supplement: Supplementary Materials — File 1: algorithm for institutional management for symptomatic pneumothorax in adult patients (≥18 yrs. of age). [file 2035248.f1.pdf]

INITIAL MANAGEMENT OF **SYMPTOMATIC ADULT (≥ 18 years)** PNEUMOTHORAX PATIENTS IN THE NON-ICU SETTING (TUALITY HEALTHCARE, Version [08/01/ 2014])[α,β]

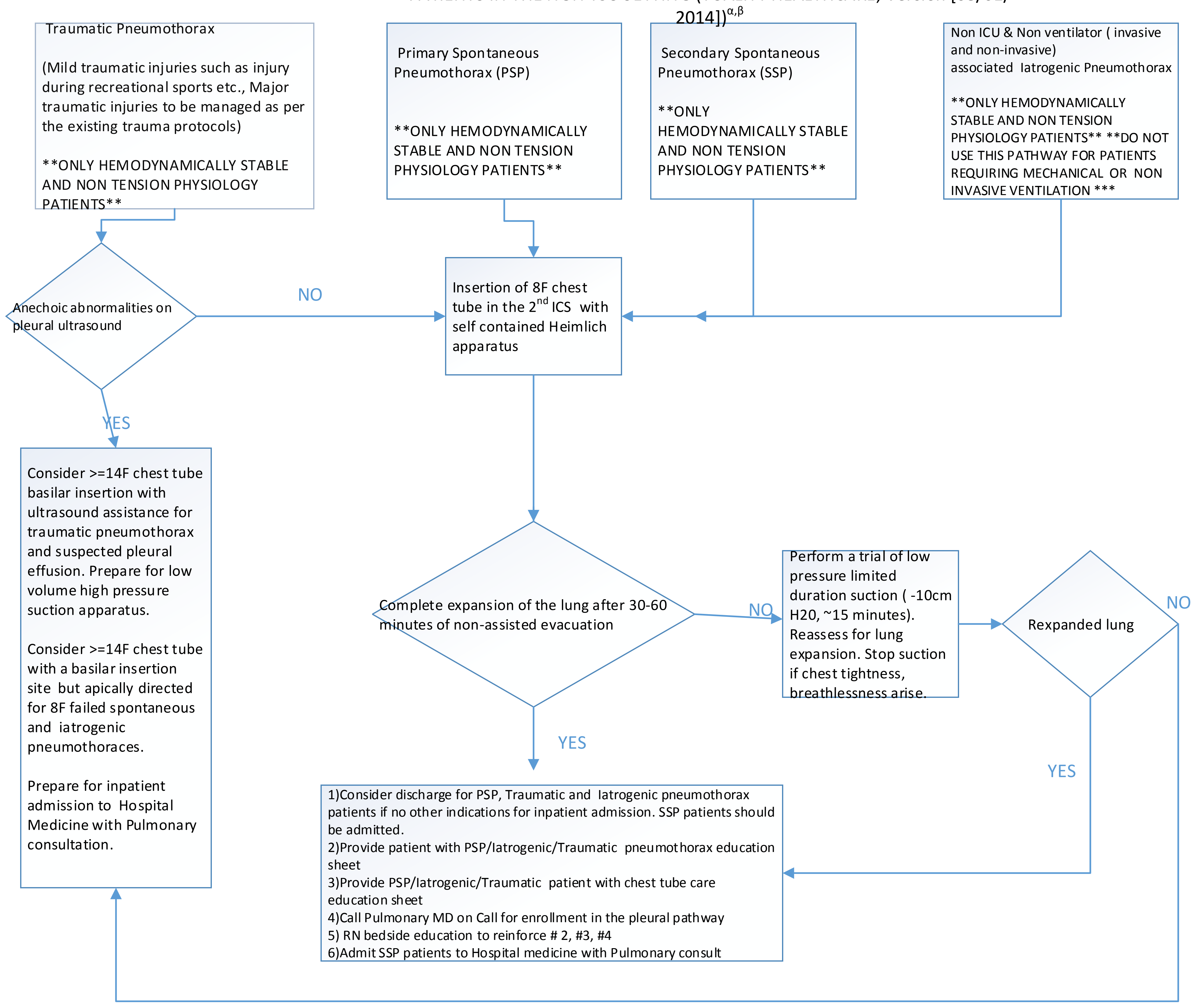

PSP: Suspect PSP in patients < 50 yrs. of age **(OR)** absence of significant smoking history **(AND)** no evidence of underlying lung disease on previous images/examination/ history

SSP: Suspect SSP in patients > 50 yrs. Of age **(OR)** presence of significant smoking history **(OR)** presence of cystic lung changes/bullae/ emphysema on previous images/ examination/history

Pulmonary consult MD **will coordinate** with other members of the pleural service such as interventional radiology, thoracic surgery for more definitive management in the outpatient and inpatient environments.

PSP without symptoms of breathlessness **AND** that is of small size (≤ 2 cm inter pleural distance at the level of hilum on imaging) can be considered for conservative management (defined as outpatient discharge without 8F chest tube insertion, return to ER if symptoms arise and assured follow up in the pulmonary clinic)

Tension physiology (established or rapidly progressing towards hypotension,tachycardia,hypoxemia) will need immediate 8F chest tube/needle decompression and definitive chest tube STAT.

This document is sourced from British Thoracic Society (α) & Belgian Society of Pneumology (β) guidelines for management of spontaneous pneumothorax .These guidelines have been modified to suit unique local needs , resources, workflows.

α MacDuff A, Arnold A, Harvey J. Management of spontaneous pneumothorax: British Thoracic Society pleural disease guideline 2010. Thorax. 2010 Aug 1;65(Suppl 2):ii18-31.

β Leyn PD, Lismonde M, Ninane V, Noppen M, Slabbynck H, Meerhaeghe AV, Schil PV, Vermassen F. Belgian Society of Pneumology. Guidelines on the management of spontaneous pneumothorax. Acta chirurgica Belgica. 2005 Jan 1;105(3):265-7.
